# Osmotic demyelination syndrome (ODS), and psychiatric manifestations

**DOI:** 10.1192/j.eurpsy.2024.996

**Published:** 2024-08-27

**Authors:** P. Argitis, A. Karampas, M. Peyioti, A. Goudeli, S. Karavia, Z. Chaviaras

**Affiliations:** ^1^Psychiatric, General Hospital of Corfu, Corfu; ^2^Psychiatric, General Hospital of Ioannina, Ioannina, Greece

## Abstract

**Introduction:**

Hyponatriemia can be potentially fatal if it is not corrected immediately. The rapid correction of chronic hyponatriemia can cause demyelinating brain lesions.

**Objectives:**

A fifty-six year old female was brought to the emergency department of the psychiatric clinic by her daughter, with incomprehensible speech and psycomotor agitation. She was diagnosed several years ago with bipolar disorder, with valproic acid and quetiapine being her current medication. She has been living alone, in a small suburban city. Approximately twenty four hours before her admission to the hospital she visited her daughter, which aligns with the onset of symptoms.

**Methods:**

After both the brain CT scan and the lab results came back normal, the patient was admitted to the psychiatric clinic of the General Hospital of Corfu. On the fourth day of the patient’s hospitalization - when both her speech and the psycomotor agitation showed signs of improvement- we were informed that three days before her admission to the clinic she visited the emergency department of another hospital where she was treated for hyponatriemia. The patient’s hyponatriemia was corrected over the span of twelve hours by 35 mEq.

**Results:**

After receiving this information, we ordered a brain MRI scan which revealed a central pontine myelinolysis. The result can explain the clinical symptoms that our patient showcased before her admission and could have been caused by the rapid correction of hyponatriemia.

**Conclusions:**

The patient’s speech was fully restored after four weeks and there were no symptoms consistent with any psycho emotional disorder.

**Disclosure of Interest:**

None Declared

